# The status of pyrethroid resistance mutation frequencies in *Varroa destructor* populations in the most important beekeeping areas of Türkiye

**DOI:** 10.1007/s10493-025-01002-0

**Published:** 2025-01-28

**Authors:** Elif Celikkol, Ersin Dogac

**Affiliations:** 1 Institue of Science, Department of Molecular Biology and Genetics, Mugla Sıtkı Koçman University, Mugla, Türkiye; 2 Faculty of Science, Department of Molecular Biology and Genetics, Mugla Sıtkı Koçman University, Mugla, Türkiye

**Keywords:** *Varroa destructor*, Insecticide resistance, PCR-RFLP, *Vgsc*, Chemical control

## Abstract

The *Varroa destructor* (hereafter referred to as *Varroa*) is a major pest of honeybees that is generally controlled using pyrethroid-based acaricides. However, resistance to these insecticides has become a growing problem, driven by the acquisition of knockdown resistance (*kdr*) mutations in the mite’s voltage-gated sodium channel (*vgsc*) gene. Resistance mutations in the *vgsc* gene, such as the L925V mutation, can confer resistance to pyrethroids like flumethrin and tau-fluvalinate. Monitoring genotypic resistance through molecular mutation screening is crucial for tracking and mitigating resistance spread. In this study, the frequency of resistance mutations in the *vgsc* was examined using a Polymerase Chain Reaction-Restriction Fragment Length Polymorphism (PCR-RFLP) approach in *Varroa* populations sampled throughout the Mediterranean, Aegean, and Black Sea regions of Türkiye. Considering all the samples analyzed, the results demonstrated a mean resistance allele frequency of 83.29%, indicating a relatively high frequency of resistant alleles. We observed 94.58%, 85.71%, and 69.58% resistant allele frequencies in populations sampled from the Mediterranean, Aegean, and Black Sea regions, respectively, in our study. The results of our investigation demonstrated substantial regional variations in the frequencies of resistant alleles among *Varroa* populations throughout Türkiye, with notably elevated resistance levels observed in the Mediterranean and Aegean regions. Due to the significant resistance mutation frequency differences between both provinces and regions, long-term monitoring of resistance alleles and the planning of regional control strategies are required for effective control of this pest.

## Introduction

Since ancient times, honey has been considered the primary product that comes to mind when discussing the outputs of beekeeping. Türkiye is one of the oldest and most popular apiculture and honey production hubs worldwide because of its geographic location, rich flora, variety of vegetation types, and climate (Kekecoglu et al. 2007). Türkiye ranks second in the world in terms of bee product output, with 95,386 companies, 8,984,676 hives, and annual honey production of approximately 114,886 tons (TÜİK 2023). In addition to honey and other bee products, apiculture has been one of the most significant activities for human civilization, from antiquity to the present, because of its crucial role in plant pollination. The importance and value of pollination have reached unprecedented levels in contemporary times. Pollination benefits 35% of the human diet (Klein et al. 2007). Over the years, pollinators, both wild and managed, such as the western honey bee Apis mellifera, have been able to effectively meet this growing need (Garibaldi et al. 2017). However, if wise and practical actions are not taken, a multitude of interrelated pressures on these species have warned of an impending ecological and economic catastrophe (Potts et al. 2010).

*Varroa*, a ubiquitous ectoparasitic mite whose natural host is *Apis cerana*, is the primary pest in apiculture. It feeds on the fat bodies of adult honeybees (*Apis mellifera*) and broods, contributing to their mortality and significantly increasing colony mortality rates (Anderson and Trueman 2000; Ramsey et al. 2019). *Varroa* entered Türkiye in 1976 when it was transported to the Thrace region via Bulgaria and spread all over the country in a very short period of 4–5 years through itinerant beekeeping activities (Akyol and Korkmaz 2005). Türkiye’s geography, climate, honeybee trade, and nomadic beekeeping practices have accelerated *Varroa*’s spread (Warrit et al. 2004; Koç et al. 2021). Similar to many beekeepers worldwide, beekeepers rearing *A. mellifera* in Türkiye are trying to limit the parasite pressure in their hives with various control strategies against *Varroa* infestation (Rosenkranz et al. 2010).

These mites not only feed on honey bees and inflict direct physical harm, but they also deplete honey bee nutrition stores and serve as carriers of multiple dangerous viruses, such as the Israeli acute paralysis virus, the deformed wing virus, and the acute bee paralysis virus (Lee et al. 2023). Because of these adverse effects, these mites are often acknowledged as one of the main causes of colony collapses (Le Conte et al. 2010). Inadequate mite control measures can cause rapid colony collapses, severely reducing bee populations (Rosenkranz et al. 2010). As a result, controlling *Varroa* in apiaries is crucial to preserving the health of the colonies.

A recent study (Vaes-Petignat and Nentwig 2014) found that *Varroa* is the most harmful invasive arthropod species in Europe, and actions need to be taken to ensure that beekeeping continues to exist globally. Beekeepers had to employ a number of synthetic or organic acaricides to manage the *Varroa* infestation (Roth et al. 2020). Among the different synthetic acaricides, tau-fluvalinate and flumethrin were the most commonly used varroacides, which have highly selective toxicity to *Varroa*, causing minimal harm to honey bees (Vlogiannitis et al. 2021). Members of the pyrethroid class, such as tau-fluvalinate and flumethrin, disrupt the *vgsc* activity, which affects the nervous system of mites (Taskin et al. 2011, 2016; Millán-Leiva 2022). Unfortunately, the widespread use of these acaricides has resulted in the development of resistance to them, which makes control failures frequent (Higes et al. 2020). The recent rapid rise in knockdown resistance (*kdr*) to pyrethroid insecticides worldwide, which is inherited as a recessive trait, has made it difficult to control *Varroa* using pesticides. The intensive and widespread use of pyrethroids, which alter the gating kinetics of voltage-sensitive sodium channels, exerts a strong selection pressure on populations. *Kdr*, which results in a change in affinity between the insecticide and the binding site on the sodium channel, is caused by single or multiple point mutations in the channel gene (Hemingway et al. 2004). The majority of these point mutations are found in the IIS4-S5 linker and in the domain II and III transmembrane helices (IIS5-IIS6 and IIIS6). According to modeling studies, pyrethroids will bind to these locations, which have the structure of a hydrophobic pocket (Millán-Leiva 2022). Amino acid substitutions in the *vgsc* protein have been associated with reduced susceptibility to pyrethroids in *Varroa* populations. In *Varroa* (*kdr*-type resistance) populations, substitutions of L925V, L925I, L925M (Gonzalez-Cabrera et al. 2013, 2016, 2018), and L918M (Millan-Leiva et al. 2021) have been found and associated with pyrethroid insensitivity. The prevalence of these substitutions seemed to increase over time, likely because of selection pressure from the use of pyrethroids.

Laboratory assays, which were first developed by Milani (1995), are often used to determine whether *Varroa* are resistant or susceptible (Kamler et al. 2016). The evaluation process for these biological tests is rather traditional, laborious, complex, and prone to multiple sources of mistake. In addition, the majority of laboratories cannot afford biomolecular procedures like TaqManVR. Nonetheless, Millan-Leiva et al. (2018) showed that a novel PCR-RFLP methodology was simpler and less expensive than Taqman-based high-throughput genotyping while still being as accurate and reliable. Thus far, this technique has successfully evaluated pyrethroid resistance by identifying pyrethroid target-site resistance mutations at *vgsc* position 925.

**Fig. 1:**
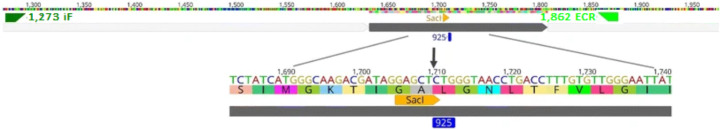
Illustration of the SacI restriction site (5′-GAGCT/C-3′) and the PCR-amplified region of the *Varroa* vgsc Domain II. (adapted from Millan-Leiva et al. 2018)

Mutations identified at position L925 of the *Varroa* vgsc protein (Gonzalez-Cabrera et al. 2013, 2016, 2018; Hubert et al. 2014; Alissandris et al. 2017; Millan-Leiva et al. 2021) resulted from non-synonymous substitutions at position 1710. These substitutions were G/C, leucine (CTG) to valine (GTG) or A/C, leucine (CTG) to methionine (ATG), and 1712: A/C and A/G, leucine (CTG) to isoleucine (ATA) at positions 1710 and 1712, respectively. Sequence analysis identified a SacI restriction enzyme recognition site (GAGCT/C) between positions 1709 and 1710 (Fig. 1). Consequently, digesting a DNA fragment that encompasses the region corresponding to position 925 of the channel protein with SacI would facilitate the differentiation of the three potential phenotypes (SS, SR, and RR).

In this study, PCR-RFLP was employed to analyze *Varroa* populations collected from hives treated with flumethrin strips. Samples were collected from Muğla, Aydın, İzmir, Ordu, Trabzon, Kastamonu, Adana, Antalya, and Mersin provinces in the Aegean, Black Sea, and Mediterranean regions of Türkiye, where apiculture is most prevalent. The objective was to examine the mutations at position 925 of the *vgsc* gene associated with pyrethroid resistance and to evaluate the prevalence and intensity of resistance levels in these populations. To address the pressing issue of resistance in the *Varroa* populations outlined above, this study employed the PCR-RFLP methodology to examine mutations in samples collected from key beekeeping provinces in Türkiye.

## Materials and methods

### Sampling of *Varroa* mites

**Fig. 2:**
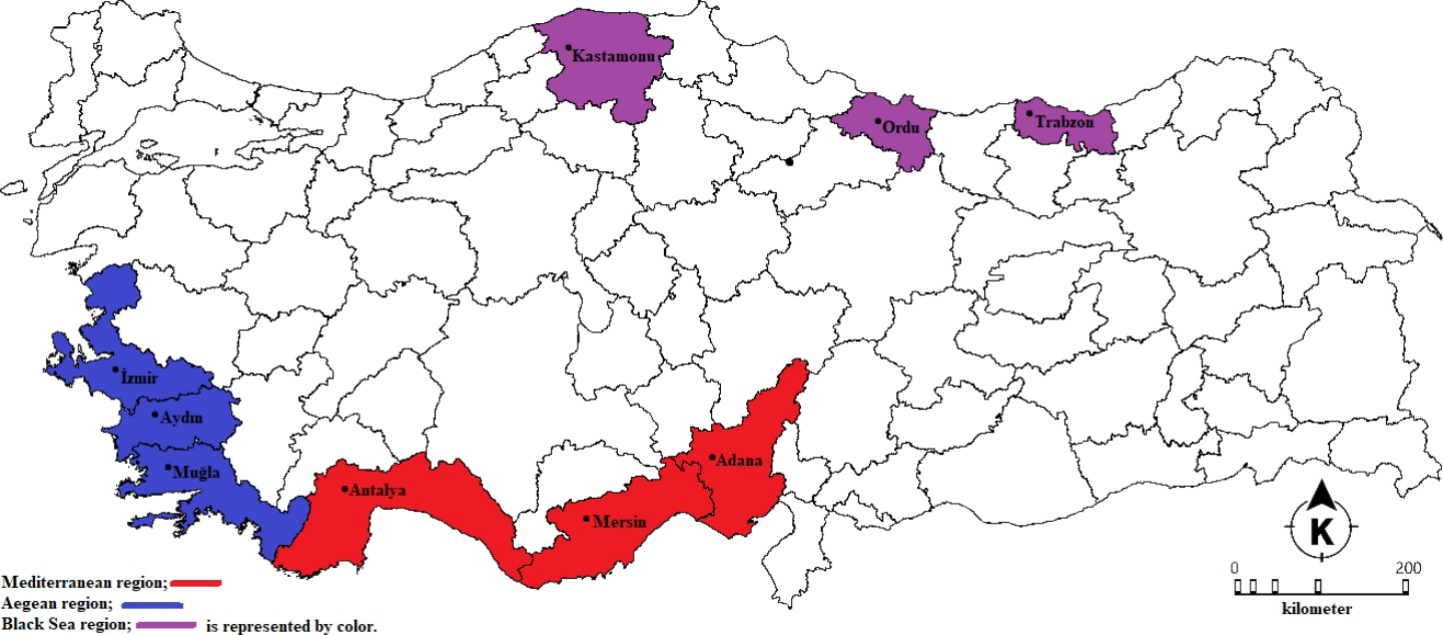
Collection sites of *Varroa* samples. (Mediterranean region; red, Aegean region; blue and Black Sea region; purple)

In September 2022, female mites were sampled from hives where flumethrin strips (Bayvarol^®^, Bayer Healthcare AG, Türkiye) were used in Muğla, Aydın, İzmir, Ordu, Trabzon, Kastamonu, Adana, Antalya, and Mersin provinces of the Aegean, Black Sea, and Mediterranean regions of Türkiye (Fig. 2). Sampling was carried out to characterize the provinces where beekeeping is most intensive in Türkiye. *Varroa* samples were collected from worker bees using a fine paint brush in accordance with the manual collection procedure described by Dietemann et al. (2013), with 10 specimens from each hive and 80 specimens from each province. The hives used for sampling were selected from areas without nearby beekeepers, to avoid interference or external influences. Sampling was conducted from the hives belonging to students of Muğla Sıtkı Koçman University Ula Vocational School Beekeeping Program under the supervision of Dr. Taylan DOĞAROĞLU from hives where flumethrin strips were utilized. Samples were fixed in 80% ethanol, moved to pure ethanol under a dissecting microscope, and stored until DNA extraction.

### Amplification and PCR-RFLP assay of the *vgsc* fragment

Genomic DNA of samples collected from the nine provinces (80 from each province, a total of 720 individuals) was obtained from each female individual using the Zymo Research Quick DNA Miniprep Plus Kit (Cat. No.: D4069). In this study, the base sequence of the *Varroa vgsc* gene in GenBank was used as a template (Accession Number: KC 152655.2). Accordingly, the forward primer 1273f (5-‘AAG CCG CCA TTG TTA CCA GA 3’) used by Millán-Leiva et al. (2018) and the alternative reverse primer ECR (5’ GTG AGA AGC GCT ACA ATG AGC 3’) designed by us were used to amplify the 925 region of the channel protein. These primers yield a 590 bp PCR product containing the mutation site in the 925 region of *Varroa*. The PCR mix contained 1 µl DNA, 12.5 µl MasterMix (Ampliqon 2X Taq MasterMix Red, Cat. No.: A180303), and 0.5 µl of each primer (10 mM) for a total reaction volume of 25 µl. The PCR conditions consisted of pre-denaturation at 95 °C for 2 min, 35 cycles of 95 °C for 30 s, 60 °C for 20 s, 72 °C for 1 min, and a final extension at 72 °C for 5 min. The PCR products obtained were run on a 1% agarose gel. Then, the amplified gene region for the detection of resistance mutation was mixed with 5 µl of PCR product, 2.5 U of the restriction enzyme SacI, and 2 µl of 10X SacI Buffer in a final volume of 20 µl and incubated at 37 °C for 1 h. The products were electrophoresed on 2% agarose gel at 100 V for 70 min.

### Interpretation of images and statistical analyses

**Fig. 3:**
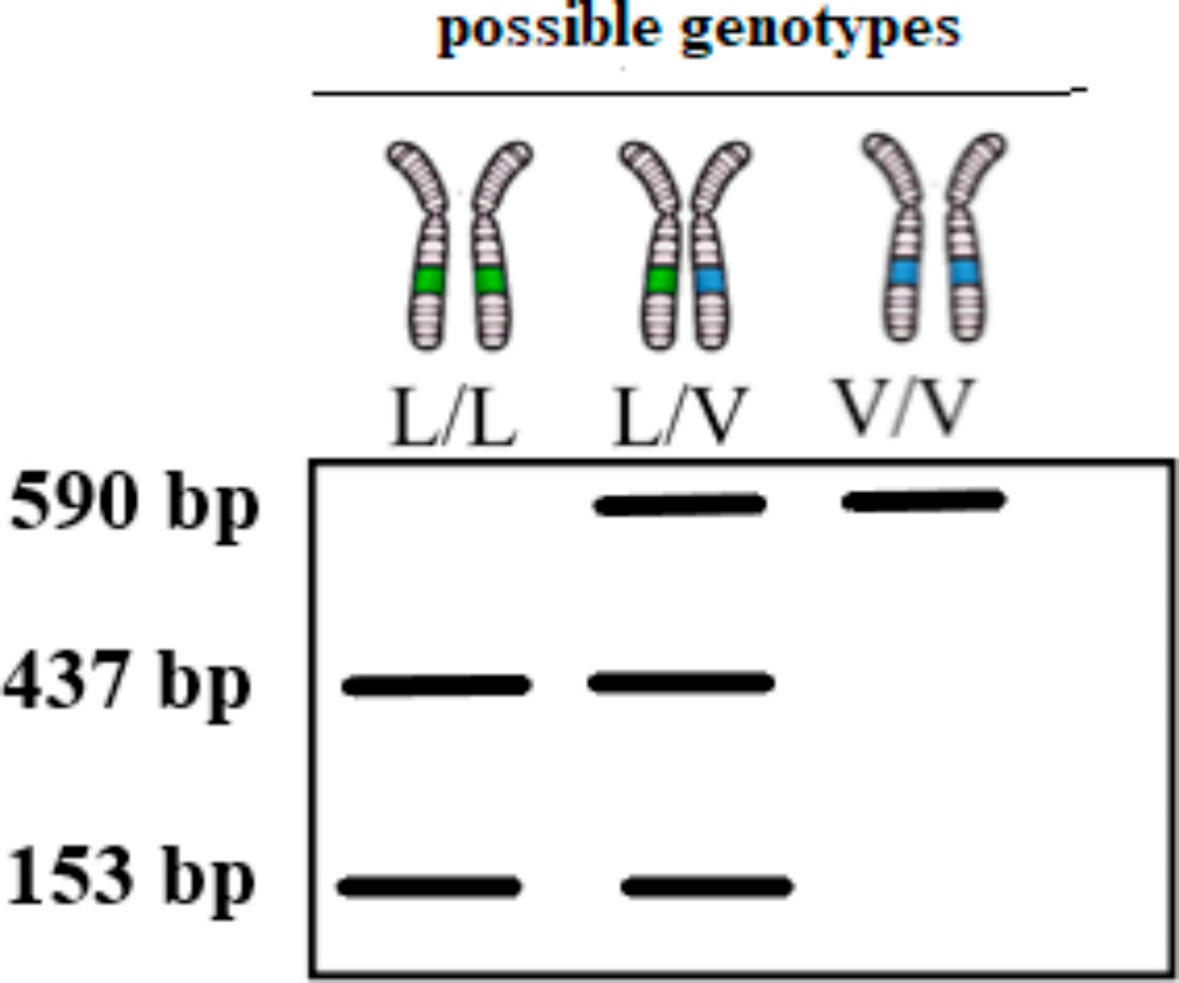
Possible genotypes for digestion of PCR fragments with the SacI enzyme

DNA samples were utilized as templates for polymerase chain reaction (PCR) amplification of a 590 bp fragment of *Varroa* vgsc, which encompassed the 925 position. Given the presence or absence of the restriction site, digestion of the PCR fragments with the SacI enzyme produced the expected pattern after gel electrophoresis (Fig. 3). In the images obtained as a result of the analysis, individuals showing a double-banded pattern (437 and 153 bp) were characterized as homozygous (SS) for the susceptible allele, individuals showing a single 590 bp band were characterized as homozygous (RR) for the resistant allele, and individuals showing a triple-banded pattern (590, 437, and 153 bp) were characterized as heterozygous (RS). Because *kdr* and *super-kdr* resistance traits are recessively inherited, resistant mites are only found in lanes that have a single 590-bp band (Davies et al. 2007).

Prior to analyses, the resistance frequencies data were examined using the Shapiro-Wilk and Bartlett tests to determine whether each met the parametric test requirements. One-way ANOVA and Tukey’s HSD test or Kruskal-Wallis and Dunn’s test were applied to parametric and nonparametric data, respectively, to compare differences among provinces. The significance level was set at 0.05 for all analyses.

## Results

**Fig. 4:**
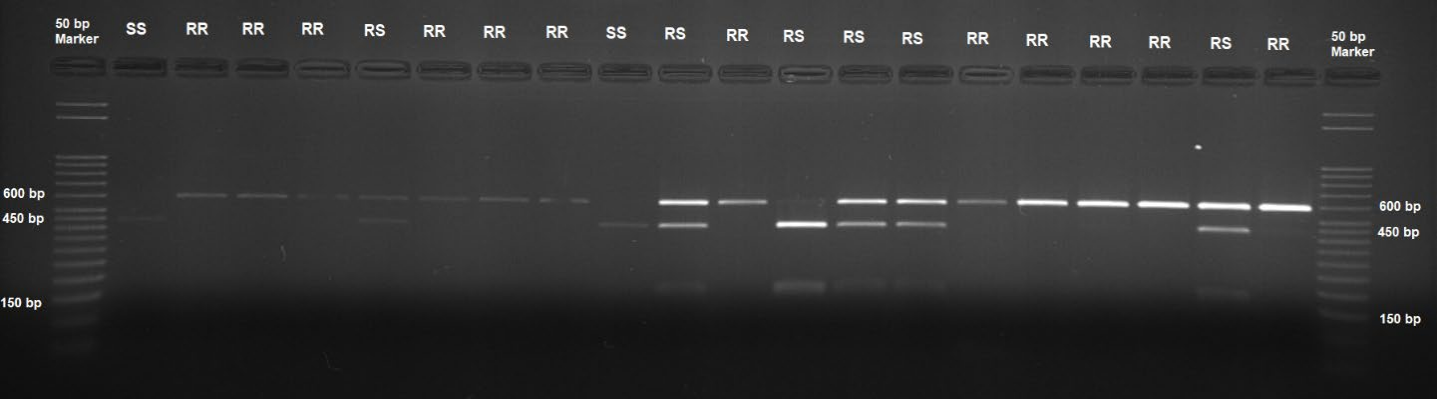
Restriction enzyme digestion profile of PCR product sampled from Trabzon province

This study investigated the presence of mutations at position 925 in the IIS5 region of the *vgsc* gene, which is associated with resistance to pyrethroids in *Varroa*, utilizing the PCR-RFLP method. To this end, 718 *Varroa* samples collected from three distinct geographical regions and nine different provinces in Türkiye were subjected to digestion with SacI. Although this study investigated *Varroa* samples with susceptible or resistant alleles, the findings can also be applied to nearly all alterations of the first nucleotide of the first nucleotide of the CTG codon that codes for L925 (Fig. 1). Thus, mites homozygous for the susceptible allele (L/L) or heterozygous for the susceptible allele (L/I or L/M) generate the identical two or three band patterns as seen in Fig. 3. Conversely, mites homozygous for the resistant alleles (I/I or M/M) or heterozygous without the susceptible allele (M/I, V/I, or M/V) exhibit a single 590 bp band when analyzed on a gel. As previously mentioned, the presence of a solitary 590 bp band in the DNA indicates resistant mites in these instances (Fig. 4).

**Fig. 5:**
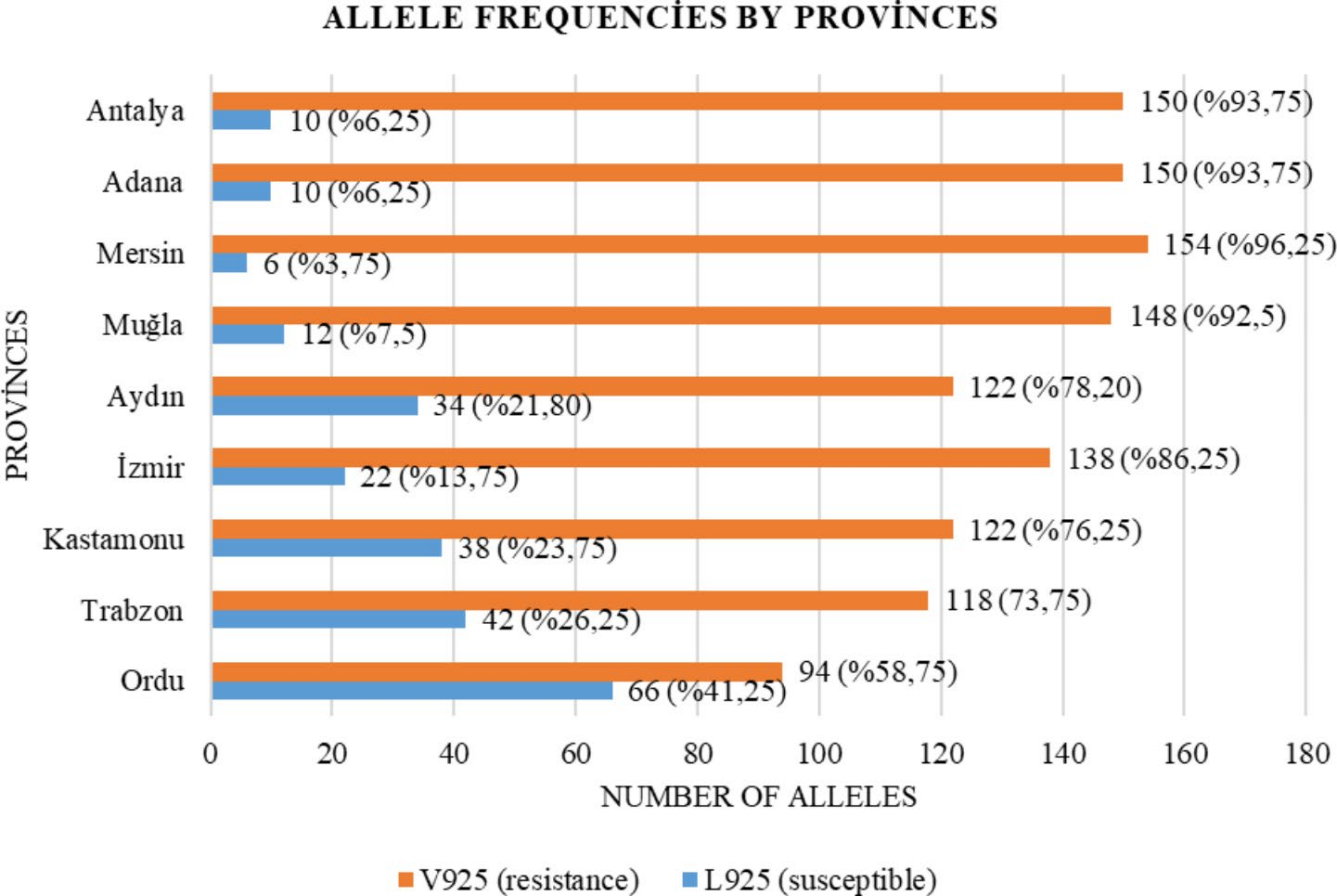
Distribution of allele numbers and frequencies by province. Susceptible alleles are represented in blue, whereas resistant alleles are indicated in orange

Of the 718 samples analyzed, 498 (69%) were homozygous resistant, 200 (28%) were heterozygous susceptible, and 20 (3%) were homozygous susceptible genotypes. No homozygous susceptible (SS) individuals were detected among the mites collected from Adana, Mersin, Antalya, Muğla, İzmir, and Aydın provinces (Table 1; Fig. 5).

**Table 1:** Distribution of pyrethroid resistance in *Varroa* mites according to provinces

Provinces	Sample size	Number of homozygous resistant individuals (RR)	Number of heterozygous resistant individuals (RS)	Number of homozygous susceptible individuals (SS)
Adana	80	70	10	0
Mersin	80	74	6	0
Antalya	80	70	10	0
Muğla	80	68	12	0
Aydın	78	44	34	0
İzmir	80	58	22	0
Trabzon	80	48	22	10
Ordu	80	20	54	6
Kastamonu	80	46	30	4
Total	**718**	**498**	**200**	**20**

According to the allele frequencies, the provinces with the highest resistant allele frequency are Mersin with 96.25%, Antalya and Adana with 93.75%, and Muğla with 92.5%. The provinces with the highest susceptible allele frequencies are Ordu, Trabzon, and Kastamonu, with 41.25%, 26.25%, and 23.75%, respectively (Table 1; Fig. 3).

When the analyzed samples were compared on the basis of regions, *Varroa* sampled from the Mediterranean region ranked first in resistance to pyrethroid insecticides, with 214 homozygous resistant individuals and a 94.58% resistant allele frequency. The mites sampled from the Aegean region with 170 homozygous resistant individuals and 85.71% resistant allele frequency, followed by the mites sampled from the Black Sea region with 114 homozygous resistant individuals and 69.58% resistant allele frequency, ranked third in terms of pyrethroid resistance. In Türkiye, the mean frequency of overall resistant alleles is 83.29%, whereas the frequency of susceptible alleles is 16.71%.

The provinces in the Aegean and Black Sea regions met the parametric test conditions and underwent an ANOVA test for statistical analysis. While the difference in resistance mutation frequency between the provinces in the Aegean region was not statistically significant (*p* = 0.158), the difference in resistance mutation frequency was found in the provinces in the Black Sea region (*p* = 0.042), and a parametric Tukey’s HSD multiple comparison test was applied to these provinces. The analysis revealed that the difference in resistance mutation frequency between Ordu and Kastamonu was statistically significant (p˂0.05). In the Mediterranean region, the Kruskal-Wallis test was used since parametric test conditions were not met and the difference in resistance mutation frequency between the provinces was not found to be significant (*p* = 0.625). The statistical analysis results for the Aegean and Black Sea regions are presented in Table 2.

**Table 2:** Analyses of the provinces in the Mediterranean, Black sea and Aegean regions

Aegean region		Black sea region		Mediterranean region	
Provinces	Mean ± St. Dev.	Provinces	Mean ± St. Dev.	Provinces	Mean ± St. Dev.
**Muğla**	0,925 ± 0,060^a^	**Kastamonu**	0,762 ± 0,055^a^	**Antalya**	0,0938 ± 0,031^a^
**Aydın**	0,762 ± 0,069 ^a^	**Trabzon**	0,738 ± 0,024^ab^	**Mersin**	0,963 ± 0,013 ^a^
**İzmir**	0,863 ± 0,024 ^a^	**Ordu**	0,588 ± 0,047^b^	**Adana**	0,938 ± 0,063 ^a^

Values with different superscript letters in the same row are significantly different from each other (*p* < 0.05; Tukey’s HSD test)

The highest resistance mutation frequency was found in the Mediterranean region. However, there was no statistically significant difference between the Mediterranean and Aegean regions, while the Black Sea region had a significantly lower resistance mutation frequency difference compared to the other two regions (Table 3).

**Table 3:** Results of statistical analysis (one-way ANOVA) of resistance mutation frequency by region (*p* = 0,0001)

Regions	Mean ± St. Dev.
**Mediterranean**	0,946 ± 0,022^a^
**Aegean**	0,850 ± 0,035^a^
**Black Sea**	0,696 ± 0,033^b^

Values with different superscript letters in the same row are significantly different from each other (*p* < 0.05; Tukey’s HSD test)

## Discussion

The intensive and uncontrolled use of pyrethroids in the chemical control of *Varroa* has led to the development of resistance in many mite populations worldwide (Alissandrakis et al. 2017; Farjamfar et al. 2018; González-Cabrera et al. 2018; Panini et al. 2019; Stara et al. 2019; Higes et al. 2020; Koç et al. 2021; Millán-Leiva et al. 2021; Vlogiannitis et al. 2021; Hernández-Rodríguez et al. 2022; Morfin et al. 2022; Lee et al. 2023; Erdem et al. 2024; Li et al. 2024; Yarsan et al. 2024).

Since the early 1990s, there have been reports of pyrethroid resistance in *Varroa* throughout Europe (Martin 2004; Koç et al. 2021). However, in Türkiye, there is one commercially licensed drug, Apistan 10%, with the active ingredient tau-fluvalinate, and four different licensed drugs, Bayvarol, Flumevar, Varostop, and Beevarflu, with the active ingredient flumetrin (Özdemir and Muz 2021). In this study, we investigated the presence of mutations occurring at position 925 of *vgsc* that cause resistance to pyrethroids in *Varroa* and the distribution of these mutations in Türkiye. As a result of PCR-RFLP analyses, mutations at position 925 were found in all populations sampled, which provides resistance and reflect the widespread use of pyrethroids.

Previous studies conducted in Türkiye show that resistance mutations in the *vgsc* gene are common, in parallel with the results obtained in our study. Koç et al. (2021) reported the identification of resistance mutations in over 75% of all populations across 17 locations (one from Ordu, one from Muğla, one from Eskişehir, one from Zonguldak, and the remainder from Ankara), including Ordu and Muğla provinces, although they did not specify the resistance levels of the locations. According to Erdem et al. (2024), approximately 80% of 44 *Varroa* populations sampled from 21 provinces, including only Ordu province, showed resistance mutations. The researchers reported that the resistance levels in two colonies from Ordu were 33.9% in colonies treated with flumetrin and amitraz (ORD1) and 100% in colonies treated with amitraz, organic acid, and essential oil. Finally, Yarsan et al. (2024) investigated resistance levels in samples obtained from seven distinct locations subjected to pyrethroid treatment. Their findings, which encompassed only Ordu and Muğla provinces, indicated that the resistance levels ranged from 51 to 94%, with specific resistance levels of 89% for Muğla (18 samples) and 94% for Ordu (32 samples). In the present study, the frequency of the resistance allele ranged from 58.75 to 96.25% among the provinces analyzed. When evaluated based on the same provinces in previous studies, resistance levels of 92.5% for Muğla and 58.75% for Ordu were determined in our investigation. The difference in resistance levels seems to be probably due to differences in sample numbers/colonies or application differences. Consequently, it is imperative to examine the resistance levels of these provinces with a larger sample size and diverse colonies. The findings obtained in all these studies emphasize the need to reconsider the management and control strategies of insecticide resistance in Türkiye. For example, controlling these mites through the rotational use of pyrethroids with organic acids and other chemical control options like amitraz or coumaphos may decrease the frequency of resistance mutations and enable a more long-lasting chemical control approach. In addition, monitoring the resistance status in mite populations by molecular analyses such as PCR-RFLP is particularly important for early detection of resistance on a global scale. This method, which we used in our study, is not costly and can be performed in almost any laboratory. The continued use of pyrethroids with the active ingredient flumetrin by beekeepers in Türkiye to control *Varroa* may also lead to the fixation of resistance alleles.

In this study, only 19.6% of 478 samples were heterozygous in the Mediterranean and Aegean regions. Similar to our findings, Alissandrakis et al. (2017), Panini et al. (2019), Mitton et al. (2021), Almecija et al. (2022), and Li et al. (2024) reported that the rates of heterozygous individuals were 8.9%, 2%, 23%, < 3% and 12.5%, respectively. The high inbreeding and haplodiploid modes of inheritance in *Varroa* account for the low frequency of heterozygous individuals. However, considering that *kdr*-*type* resistance is a recessive trait and therefore heterozygous genotypes are phenotypically susceptible to pyrethroids, the low number of heterozygotes in mite populations may also be due to the fact that the mutant mites surviving after pyrethroid treatments are homozygous. Only mites homozygous for the resistant gene are phenotypically resistant to pyrethroids, since *kdr*-*type* resistance is a recessive characteristic (Benito-Murcia et al. 2022). Beaurepaire et al. (2017) reported that heterozygous genotypes were more frequently detected in seasons with low offspring numbers, such as fall or early winter. These seasonal fluctuations can make important contributions to the control of *Varroa*. For example, pyrethroid group insecticides used in the control of these mites can be applied when the number of heterozygous individuals is at its highest. The increased resistance of these mites to insecticides poses an even greater threat to the survival of honeybee colonies. For example, Bak et al. (2012) reported in a study conducted in Poland that failure to detect mite resistance to pyrethroid insecticides early enough led to 75% mortality in honey bee colonies in winter.

In our study, 94.58%, 85.71%, and 69.58% resistant allele frequencies were observed in populations sampled from the Mediterranean, Aegean, and Black Sea regions, respectively. Similarly, Panini et al. (2019) reported in their study conducted in Italy that resistance rates were higher in the southern regions, while resistance rates remained at lower levels in the northern regions. Additionally, the accelerated population growth of *Varroa* mites in the Mediterranean climate compared to temperate regions is attributed to the presence of continuous broods, which contribute to the development of resistance (Kraus and Page Jr 1995). This situation, as observed in our study, shows that resistance is not spread homogeneously among regions and that different chemical usage plays an important role in resistance development. It also suggests that climatic conditions may contribute to resistance development (Kraus ve Page Jr 1995; Taskin et al. 2016; Beaurepaire et al. 2017; Maino et al. 2018).

Considering the current situation in all of the populations analyzed in our study, the frequency of resistant alleles was quite high, and the mean frequency of overall resistant alleles was 83.29% in all samples evaluated. Based on this result, it seems that the successive use of pesticides with flumetrin active ingredients in the provinces where we sampled significantly triggers resistance and increases the rate of resistant mites. However, the acquisition of resistance is usually associated with disadvantages in the survival and reproductive abilities of mites, and the frequencies of resistance alleles in the population decrease if the insecticide with the relevant active ingredient is not used. This phenomenon is called reversion and is observed when insecticide application is stopped or reduced (Thompson et al. 2002). Therefore, discontinuing the use of pyrethroids in the management of *Varroa* mites in populations where resistance has been observed may result in a reduction in resistance allele frequencies over time. For instance, research conducted by Milani and Della Vedova (2002) in northern Italian apiaries, where pyrethroids were not utilized, revealed a significant decrease in resistant mite populations over a three-year period from 1996 to 2000. The study observed that the proportion of resistant mites declined by approximately 10-fold during this period. González-Cabrera (2018) examined the correlation between insecticide use and shifts in genotype frequencies. The findings showed that when a colony typically treated with Apistan was left untreated for 8 months, the percentage of susceptible (SS) genotypes in the sampled population increased from 34.6 to 70.8%. Similarly, Almecija et al. (2022) reported that the proportion of mites with susceptible genotypes in *Varroa* populations that had not been subjected to tau-fluvalinate treatment for more than two years was 97%, suggesting that *Varroa* mites can regain their susceptibility to tau-fluvalinate relatively rapidly.

Given the economic importance of beekeeping, information on pyrethroid resistance mutations in *Varroa* populations in Türkiye is very limited. This study reveals the status of pyrethroid resistance mutations and regional differences in *Varroa* populations in Türkiye. Monitoring these mutations will contribute to the development of effective control programs in the long term. In general, beekeepers are becoming increasingly aware of the damage that *Varroa* infestations can cause, and the evolution of resistance poses a serious challenge to the control of these mites. This situation is particularly acute for species that have developed multiple resistances (Jack and Ellis 2021; Millán-Leiva et al. 2021; Ogihara et al. 2021). In light of this, it is crucial to apply evolutionary principles when using chemicals that maintain their efficacy in order to lessen the intensity of selection for novel resistance mechanisms. As part of a larger Integrated Pest Management (IPM) approach, for instance, the employment of techniques like the insecticide “MoA treatment windows” approach, which is advised by the IRAC and is based on the alternating of effective pesticide MoA groups, is more successful when combined with non-chemical control approaches. The main goal of the IRAC “MoA treatment windows” approach is to prevent using insecticides with the same MoA on successive generations of the target pest (Bass and Nauen 2023; Bubnič et al. 2024). Chemicals having the same MoA may be used in several applications throughout a generation or treatment “window.” Insecticides with a different MoA should be used for any further necessary treatments that fall outside of the specified treatment window. Once less than two generations have passed, a compound with a particular MoA shouldn’t be utilized again. Furthermore, the rate, method, target pest, and number of treatments for each application should all be precisely followed according to the product label (Bass and Nauen 2023; Bubnič et al. 2024).

Additionally, approaches to control should be shaped by routine monitoring of *Varroa* populations, since these populations can differ in their susceptibility to pesticides throughout their distribution. The early detection of mechanisms that undermine pesticide control is essential. This enables the implementation of measures to prevent the fixation of resistance alleles within a population (Jack and Ellis 2021; Bubnič et al. 2024). Finally, more research is needed to comprehend the ecological and evolutionary aspects that contribute to resistance formation and dissemination. This will improve the risk assessment of novel compounds and management approaches and create more precise models and forecasts of resistance evolution in *Varroa* populations (Jack and Ellis 2021; Lesler 2023; Bubnič et al. 2024). Concurrently, a better understanding of the pleiotropic impacts of these resistance mutations on Varroa fitness will facilitate more efficient pest management.

## Data Availability

The datasets generated during and/or analysed during the current study are available from the corresponding author on reasonable request.
